# Therapeutic Plasma Exchange in a Rare Case of Metastatic Pancreatic Carcinoma Presenting as Thrombotic Microangiopathy and Acute Kidney Injury

**DOI:** 10.7759/cureus.78469

**Published:** 2025-02-03

**Authors:** Tsz Hing Mok, Chi Yuen Cheung

**Affiliations:** 1 Medicine, Queen Elizabeth Hospital, Hong Kong, HKG

**Keywords:** acute kidney injury, carcinoma, microangiopathic hemolytic anemia, plasma exchange, thrombotic microangiopathy

## Abstract

Thrombotic microangiopathy (TMA) represents a diverse group of conditions characterized by the presence of microangiopathic hemolytic anemia (MAHA), thrombocytopenia, and ischemic end-organ injury. Thrombotic thrombocytopenic purpura (TTP) is an important cause of TMA requiring urgent identification and therapeutic plasma exchange (TPE). Cancer-related TMA is commonly misdiagnosed as TTP. In contrast to TTP, there is no clear evidence supporting the routine use of TPE in TMA associated with cancers. However, there are some case reports showing that TPE may also be useful in TMA not associated with TTP, such as cancer-related TMA. In fact, there are different postulated pathophysiology leading to this disease entity. Here, we present a rare case of TMA and acute kidney injury (AKI) secondary to metastatic pancreatic carcinoma who had a resolution of bleeding symptoms with complete hematological and renal recovery after four sessions of daily TPE. The patient finally died of cancer six months later. Our case shows that TPE could be valuable in cancer-related TMA in particular patients, especially those with clinical bleeding and thrombosis. The ability of TPE to remove abnormal components in the complement pathway and potential endothelial-damaging agents or autoantibodies and replace them with normal ones may explain its usefulness in some patients with cancer-related TMA.

## Introduction

Thrombotic microangiopathy (TMA) is a clinical condition characterized by the presence of microangiopathic hemolytic anemia (MAHA), thrombocytopenia in conjunction with an elevated lactate dehydrogenase (LDH) level, reticulocytosis, and hyperbilirubinemia. The clinical signs and symptoms are variable, which depend on the underlying diagnosis. Thrombotic thrombocytopenic purpura (TTP) is a major cause of TMA and can be confirmed by a severe reduction of activity level (<10%) of a disintegrin and metalloproteinase with a thrombospondin type 1 motif, member 13 (ADAMTS13), a von Willebrand factor (vWF)-cleaving protease, with or without the presence of antibodies to ADAMTS13 [1]. TTP is a life-threatening and acute disorder, and therapeutic plasma exchange (TPE), which can remove both anti-ADAMTS13 autoantibodies and unusually large vWF multimers, remains its standard treatment. Once diagnosed or suspected, TPE should be administered as early as possible.

On the other hand, cancer may also be rarely associated with TMA, which can be a manifestation of the cancer itself or a complication of the treatment including chemotherapy and targeted therapies. A previous study showed that the majority of these cancer-associated TMA cases were solid cancers, typically metastatic ones. Among them, lung, gastric, prostate, and breast cancers, primarily adenocarcinoma, were the most likely diagnoses, while the association between TMA and pancreatic cancer was rarely reported [2,3]. Distinguishing cancer-associated TMA from TTP can be challenging. Patients with underlying cancer may first present as TMA, which mimics TTP while the cancer itself is not clinically apparent at initial presentation. Although plasma ADAMTS13 activity levels are useful to distinguish these two entities, the results can take several days. The exact pathophysiology of cancer-related TMA remains unknown. In contrast to TTP, strong evidence supporting the use of TPE in TMA associated with cancers is lacking [1]. There have been cases showing significant hematological improvement after TPE in patients with TMA other than TTP [4,5]. While the optimal management of cancer-related TMA is still not known, TPE can be an invaluable asset in these patients, at least in life-threatening situations such as bleeding and thrombosis. Here, we report a patient presented with a clinical picture mimicking TTP who responded well with complete hematological and renal recovery after four sessions of daily TPE. She was subsequently diagnosed with metastatic adenocarcinoma of the pancreas and died six months later.

## Case presentation

A 75-year-old lady with a known history of diabetes mellitus presented to the emergency department with a sudden onset of gross hematuria and abdominal discomfort. There were no respiratory or neurological symptoms. Physical examination was unremarkable except for jaundice. There was no fever. The blood pressure was 146/77 mmHg. Routine pre-clinic blood tests three days before showed normal blood count and liver and renal function (Table 1), and she was asymptomatic at that time.

**Table 1:** Change of laboratory results before and after hospitalization ALT, alanine transaminase; LDH, lactate dehydrogenase; TPE, therapeutic plasma exchange

	Three days before hospitalization	On admission	Two days after the last session of TPE	Reference interval
Hemoglobin (g/L)	14.7	10.8	8.6	11.7-14.9
White cell count (×10^9^/L)	6.6	13.9	9.1	3.7-9.2
Platelet (×10^9^/L)	213	51	164	145-370
Reticulocyte count (%)	-	3.9	1.9	0.5-2.0
Serum potassium (mmol/L)	4.2	4.6	3.6	3.4-5.0
Serum creatinine (umol/L)	58	216	683	45-84
Serum albumin (g/L)	38	33	27	33-48
Serum bilirubin (umol/L)	23	260	29	<27
Serum ALT (IU/L)	29	3381	26	<47
Serum LDH (IU/L)	-	4159	397	110-210
Haptoglobin (g/L)	-	<0.3	0.4	0.3-2.0

**Figure 1:**
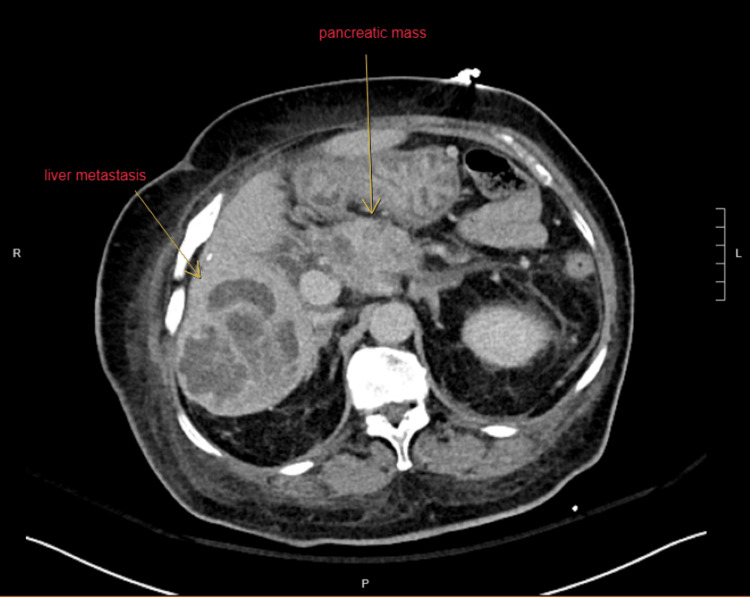
Computed tomography scan of the abdomen showing the pancreatic mass with multiple liver metastases, confirming the presence of advanced pancreatic cancer

**Figure 2:**
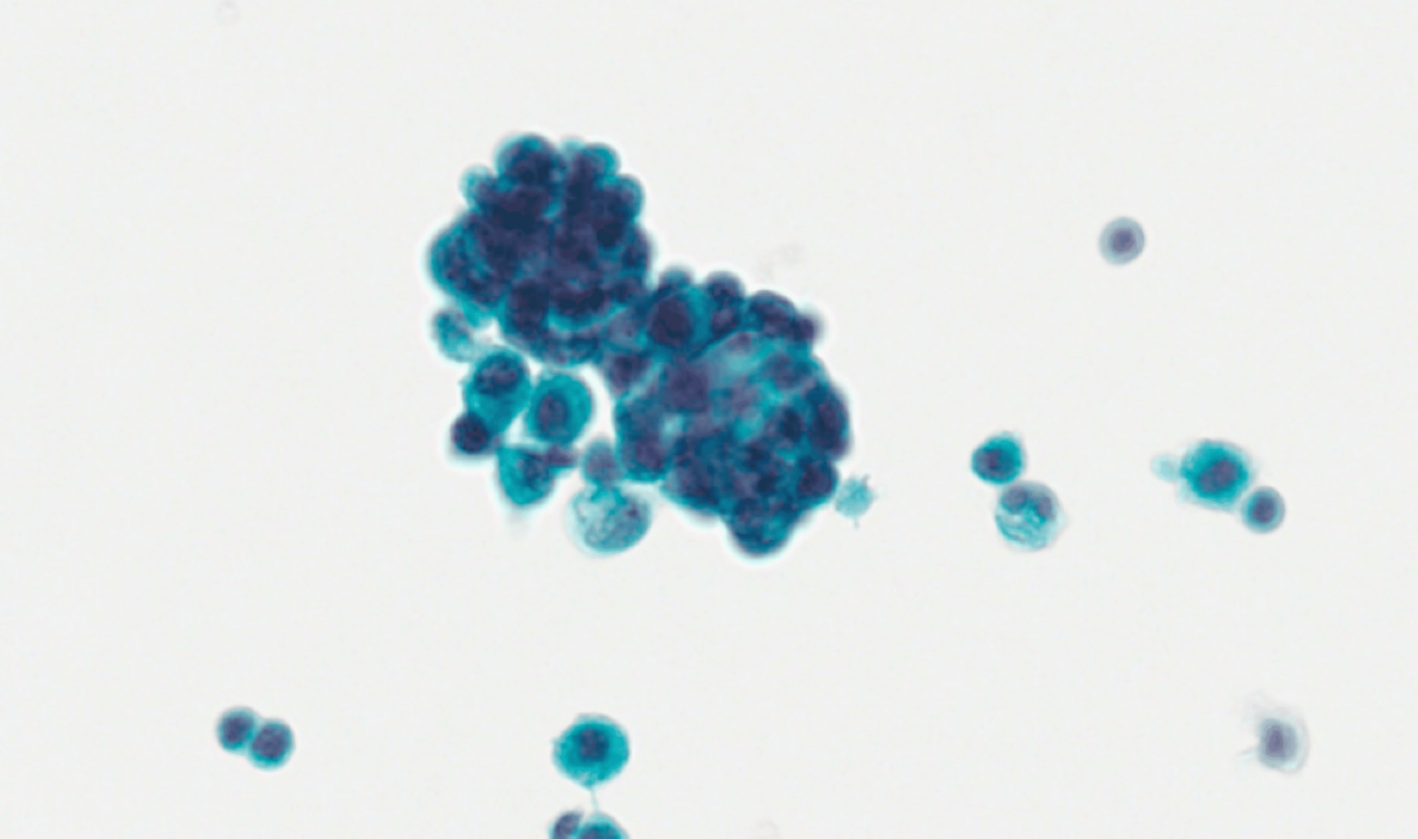
Ascitic fluid cytology showing clusters of carcinoma cells, which form irregular glandular structures with smooth communal borders, indicating malignant ascites

However, repeated blood tests after hospitalization showed normochromic normocytic anemia, thrombocytopenia, and leukocytosis. The serum LDH level was markedly elevated, while the reticulocyte count was also increased with a low haptoglobin level, indicating the presence of hemolysis (Table 1). Schistocytes were found in the peripheral blood film. The Coombs test was negative. In addition, she was also found to have acute kidney injury (AKI) and liver impairment. The prothrombin time was increased (22.2 seconds), while the activated partial thromboplastin time and plasma fibrinogen level were normal. The viral hepatitis markers and autoimmune markers were all negative. The serum complement levels (C3 and C4) were normal. The C-reactive protein was 66 mg/L. Chest X-ray was clear. Blood and urine cultures showed no growth. In view of the suspected diagnosis of TTP, daily TPE was started one day after hospitalization while still waiting for further investigation results including the ADAMTS13 test. ADAMTS13 activity level came back to be 71.9% (reference range: 60.6%-130.6%). As the ADAMTS13 result was not suggestive of TTP, TPE was stopped after a total of four sessions. Repeated blood tests showed significant improvement in blood counts and liver function (Table 1). Two days after the last session of TPE, the platelet count and the reticulocyte count were normalized. No more schistocytes were found in the blood film. However, her serum creatinine further rose up to 683 umol/L with one session of hemodialysis given. Her renal function then gradually recovered and returned to baseline 1-2 weeks later. At the same time, the cancer antigen 19.9 level was found to be markedly increased (21571 U/mL; reference range: ≤27 U/mL). A computed tomography scan of the abdomen showed a lobulated hypoenhancing soft-tissue lesion measuring around 5.0 × 3.2 × 2.8 cm involving the pancreatic head and neck region. Moreover, there were several multiloculated rim-enhancing lesions in both hepatic lobes, suggesting the diagnosis of malignant pancreatic neoplasm associated with liver metastases and ascites (Figure 1). A cytology of the ascitic fluid confirmed the diagnosis of adenocarcinoma (Figure 2). She decided to receive symptomatic treatment only after the oncologist’s assessment and finally died six months later.

## Discussion

Cancer-related TMA is a very rare cause of secondary TMA, and the incidence is only approximately 0.25-0.45 persons/million [2]. The early diagnosis of this entity is important because direct treatment of the cancers has been shown to be the key factor in improving survival in these patients to different extents [1]. Since it shares many similar clinical features with TTP such as MAHA, thrombocytopenia, and end-organ damage, distinguishing these two disease entities can be difficult. Moreover, these features, similar to TTP, may be the only initial presentation of the underlying malignancy. As our patient presented with abrupt onset of MAHA, thrombocytopenia, liver impairment, and AKI, the diagnosis was evaluated as TTP at that time. After four sessions of TPE, her clinical condition improved significantly with complete initial hematological and then renal remission. ADAMTS13 activity level came back to normal, and she was finally diagnosed to have metastatic pancreatic adenocarcinoma.

TPE was generally not advocated in patients with cancer-related TMA because of the poor clinical response reported in some previous studies and potential complications [2,3]. However, the usefulness of TPE has also been shown in a case of gastric signet ring cell carcinoma in addition to our patient [5]. In fact, it can be argued that TPE may be able to improve the acute phase of the disease to prevent early deaths so that the patients can be amenable to cancer treatment. The pathophysiology of cancer-related TMA remains largely unclear, and different mechanisms have been described. One likely cause may be the red cell fragmentation and platelet destruction in small vessels of cancerous tissues. In addition, severe injury of endothelial cells by tumor microemboli can trigger TMA in patients with cancers [6]. On the other hand, Favre et al. found that complement factor H mutations were present in two cancer-related TMA patients, suggesting the involvement of such mutations in complement cascade regulation [7]. Other than complement deregulation, interleukins may also play an important role in the development of cancer-related TMA by inducing a proinflammatory state. The interleukins can cause platelet hyper-activation and alter the red cell structure to trigger the formation of thrombi [8,9]. In such situations, the ability of TPE to remove abnormal components in the complement pathway, potential endothelial-damaging agents or autoantibodies, and replace them with normal ones may explain its usefulness in some patients with TMA other than TTP such as cancer-related TMA, complement-associated TMA, and hematopoietic stem cell transplantation-associated TMA [4,10,11].

## Conclusions

Cancer should be highly suspected in patients presenting with clinical and laboratory characteristics of TMA. Urgent chemotherapy remains the standard treatment, although the outcomes are always poor. After balancing the risks, however, TPE can still be a temporary measure in patients with clinical bleeding or thrombosis in order to prevent early deaths while waiting for definitive treatment of the underlying cause. Ideally, randomized controlled trials would be needed to recommend a specific therapy for cancer-related TMA, but the high mortality and the rarity of this condition make it difficult to perform. Alternatively, research designs such as multicenter case series would add constructiveness to our existing knowledge.

## References

[1] Scully M, Hunt BJ, Benjamin S (2012). Guidelines on the diagnosis and management of thrombotic thrombocytopenic purpura and other thrombotic microangiopathies. Br J Haematol.

[2] Lechner K, Obermeier HL (2012). Cancer-related microangiopathic hemolytic anemia: clinical and laboratory features in 168 reported cases. Medicine (Baltimore).

[3] Decaestecker A, Hamroun A, Provot F (2023). Retrospective study of 59 cases of cancer-associated thrombotic microangiopathy: presentation and treatment characteristics. Nephrol Dial Transplant.

[4] Winters JL (2017). Plasma exchange in thrombotic microangiopathies (TMAs) other than thrombotic thrombocytopenic purpura (TTP). Hematology Am Soc Hematol Educ Program.

[5] Candar O, Ekinci O, Merter M, Aslan M, Aras I (2022). Therapeutic plasma exchange in gastric signet ring cell carcinoma presenting as microangiopathic hemolytic anemia: a rare case report. J Clin Apher.

[6] Goldberg RJ, Nakagawa T, Johnson RJ, Thurman JM (2010). The role of endothelial cell injury in thrombotic microangiopathy. Am J Kidney Dis.

[7] Favre GA, Touzot M, Fremeaux-Bacchi V (2014). Malignancy and thrombotic microangiopathy or atypical haemolytic and uraemic syndrome?. Br J Haematol.

[8] Thomas MR, Scully M (2019). Microangiopathy in cancer: causes, consequences, and management. Cancer Treat Res.

[9] Bester J, Pretorius E (2016). Effects of IL-1β, IL-6 and IL-8 on erythrocytes, platelets and clot viscoelasticity. Sci Rep.

[10] Noris M, Caprioli J, Bresin E (2010). Relative role of genetic complement abnormalities in sporadic and familial aHUS and their impact on clinical phenotype. Clin J Am Soc Nephrol.

[11] Davin JC, van de Kar NC (2015). Advances and challenges in the management of complement-mediated thrombotic microangiopathies. Ther Adv Hematol.

